# Frequency of angiotensin II type 1 receptor gene polymorphism in Turkish acute stroke patients

**DOI:** 10.1111/jcmm.12017

**Published:** 2013-03-11

**Authors:** Kurt Hulyam, Bayramoglu Aysegul, Gunes Hasan Veysi, Ozbabalik Demet, Degirmenci Irfan, Colak Ertugrul, Cosan Turgut Didem, Bayram Banu, Dikmen Miris

**Affiliations:** a Department of Medical Biology, Faculty of Medicine, Eskisehir Osmangazi UniversityEskisehir, Turkey; b Department of Biology, Faculty of Science and Art, Artvin Coruh UniversityArtvin, Turkey; c Department of Neurology, Faculty of Medicine, Eskisehir Osmangazi UniversityEskisehir, Turkey; d Department of Biostatistics, Faculty of Medicine, Eskisehir Osmangazi UniversityEskisehir, Turkey; e Medical Laboratory Skills, Vocational School of Health Services, Mugla Sıtkı Kocman UniversityMarmaris, Mugla, Turkey; f Department of Pharmacology, Faculty of Pharmacy, Anadolu UniversityEskisehir, Turkey

**Keywords:** acute stroke, AT1 gene, A1166C polymorphism

## Abstract

This study was performed in acute stroke patients in the Turkish population to determine the frequency of the A1166C polymorphism in the AT1 gene and to examine the role of this polymorphism in acute stroke development. In this study, 257 genomic DNA samples were analysed (from 206 acute stroke patients and 51 healthy individuals). Genomic DNA was prepared from peripheral blood using the salt-extraction method. The presence of the A1166C polymorphism in the AT1 gene was determined using the polymerase chain reaction (PCR)-restriction fragment length polymorphism (RFLP) method. PCR products were separated by 2% agarose gel electrophoresis and visualized by a charge-coupled device (CCD) camera. In this study, the allele frequency at the A1166C position was 92% A and 8% C for control and 97% A and 3% C for patients. This difference in allele frequency between the control group and the patient group was not statistically significant. However, genotype and allele frequencies showed a significant difference (*P* < 0.001) in the control and the patient groups. The results of this study show no relationship between the A1166C polymorphism in the AT1 gene and acute stroke in the Turkish population.

## Introduction

Stroke is a common, multifactorial cardiovascular disease. A stroke event is the result of a complex interplay between traditional risk factors (*i.e*. hypertension, diabetes and smoking), environmental exposures and genetic factors 1.

The genetic basis of some metabolic and coagulation disorders predisposing to stroke is known, but the molecular basis of the genetic predisposition in the majority of stroke patients remains unknown. Genes that influence the renin–angiotensin–aldosterone system (RAAS) are potentially aetiological candidates for causing hypertension, stroke and cardiovascular disease 2.

The RAAS is important for cerebrovascular research because it influences blood pressure, vasoconstriction, thrombosis and vessel wall damage. Renin, released from the kidney, cleaves angiotensinogen (AT) to angiotensin I, which then is modified by angiotensin-converting enzyme (ACE) to angiotensin II (ATII) 3–5. Angiotensin II is a major effector of the RAAS and is involved in vasoconstriction, aldosterone secretion, renal function and cardiovascular proliferation. Two kinds of angiotensin II receptors, type 1 and type 2, have been identified, and most of the known actions of angiotensin II are mediated by the angiotensin II type 1 receptor (AT1R), which is largely associated with human cardiovascular and renal diseases. AT1R is a G protein-coupled receptor, which exerts its actions by activating phospholipase C, tyrosine kinases or non-receptor tyrosine kinases 6–8. The human AT1R (Accession Number: P30556) gene is more than 50 kb in length and contains five exons and four introns 6–9. ATR1 is expressed in different organs including the heart, skeletal muscle, brain, liver, lung and adrenal gland 5–7. Angiotensin II acts mainly *via* the angiotensin II type I receptor (AT1R) as a potent vasoconstrictor, which regulates vascular tone and systemic blood pressure 10, 11.

The A 1166 → C polymorphism of the AT1 gene has been described with either an adenine (A) or a cytosine (C) base (A/C transversion) in position 1166 of the 3′ untranslated region of the gene 9, 10, 12.

Many polymorphisms in genes of the RAAS pathway have been identified 8. AT1R A1166C has been postulated as a candidate susceptibility factor for stroke 13–15. Recent findings suggest that the A1166C polymorphism of the angiotensin II type 1 receptor gene is associated with ischaemic stroke 16.

Any possible association of the ACE and AT1R genotype with stroke pathogenesis should be important, particularly as hypertension is a major risk factor for stroke 17.

In the past few years, the genetic polymorphism (A1166C) of AT1R has been proved to be related to the stroke risk in several populations 13, 14, 18–22. However, some other studies failed to find an association between AT1R A1166C polymorphisms and stroke 23, 24. Therefore, this study was performed to determine the genotypic frequency of the A1166C polymorphism of the AT1 gene in acute stroke patients in Turkish population with the aim of examining the role of this polymorphism in stroke development.

## Materials and methods

This study included 206 acute stroke patients (94 women and 112 men; mean age 62.98 ± 12.69) and 51 controls (35 women and 16 men; mean age 57.98 ± 8.68) recruited by the medical faculty in the Neurology Department at Eskisehir Osmangazi University. Eskisehir, Turkey. The study population was genetically homogeneous and native to Turkey. Informed consent was obtained from each patient in accordance with a study protocol approved by the ethics committee of Eskisehir Osmangazi University. Acute stroke patients were separated into six subgroups according to the computed tomography (CT) and magnetic resonance imaging (MRI) results: large vessel disease (macrovascular disease), small vessel disease (lacunar infarction) cardioembolism (cardiac disease), transient ischaemic attacks (TIA), other ischaemic strokes and haemorrhage (Bleeding). Controls were selected from the individuals without personal and family history of stroke.

DNA was extracted from 10 ml of venous blood, anti-coagulated with 1.6 mg/ml ethylenediaminetetraacetic acid (EDTA) by salt-extraction method and stored at +4°C overnight. After the plasma was removed, 50 ml lysis buffer was added to the pellet. This mixture was centrifuged at 778 × g at +4°C for 15 min. Supernatant was removed and 20 ml lysis buffer was added to the pellet and centrifuged. Supernatant was removed and 5 ml natrium EDTA, 500 ml sodium dodecyl sulphate (SDS), 100 ml proteinase-K were added to the pellet. After overnight incubation at 37°C, 2 ml of NaCl was added and centrifuged at 2383 × g at +4°C for 20 min. The obtained supernatant was centrifuged at 2383 × g at +4°C for 15 min and equal volume of 2-propanol was added. DNA was seen at the end of this step. Then, the DNA was washed in the 70% alcohol and put in the vial. The alcohol was evaporated and 500 ml Tris-EDTA was added to the DNA, and incubated at 50°C overnight 25. The DNA samples were amplified using the primers, PCR mixture (one sample) and PCR conditions described below (Table 1).

**Table 1:** PCR mixture (one sample) and PCR condition

		PCR condition			
		
PCR mixture		Step	Time	Temperature (+)	Cycles
Primer 1	2 pmol	Sense: 5′- AGAAGCCTGCACCATGTTTTGAG -3′			
Primer 2	2 pmol	Antisense: 5′- CCTGTTGCTCCTCTAACGATTTA -3′			
dNTP mix	0.2 mM		4 min.	94°C	
PCR buffer		**Amplification**			30
Tris-HCl	10 mM	Denaturation	20 sec.	94°C	
KCl	50 mM	Annealing	30 sec.	62°C	
Taq pol	2 U	Extension	30 sec.	72°C	
H_2_O (distilled)	9 μl	Final extension	5 min.	72°C	
DNA	0.5–1 μg	**Hold**	–	4°C	–

**Fig. 1:**
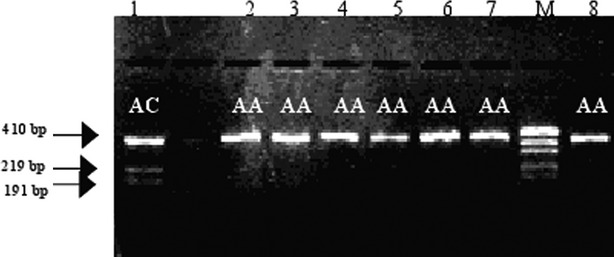
RFLP analysis of the A1166C mutation. The prototype fragment has 410 bp. The figure demonstrates M: Marker, AC: heterozygous mutant (1. sample), AA: homozygous normal (2, 3, 4, 5, 6, 7, 8, 9, 10 samples) genotypes.

PCR products, 410 bp in length, were cut by 5 U of the HindIII restriction enzyme (Boehringer Mannheim, Mannheim, Germany) according to the manufacturer's instructions. PCR fragments were separated by 2% agarose gel (63103; Sigma-Aldrich Co., St Louis, MO, USA) electrophoresis with 1 mg/ml ethidium bromide and viewed by a charge-coupled device (CCD) camera. The results were evaluated *via* gel analysis software (LabWorks, Cambridge, UK). After HindIII restriction analysis, the following three genotypes were determined: AA (410 bp), AC (410, 219, and 191 bp), and CC (219 and 191 bp; Fig. 1).

Statistical analysis was performed using the SPSS 15.0 (Statistical Package for Social Sciences) software package. Continuous variables were expressed as the means ± SE. Alleles and genotype frequencies between patients and controls were compared by the chi-squared test. Some physiological and clinical parameters of patients and controls were also analysed by the chi-squared test. The anova and chi-squared test were used to compare some physiological and clinical parameters of patients and controls, according to the AT1 gene A1166C polymorphism genotypes. *P*-values less than 0.05 were considered statistically significant.

## Results

In this study, a total of 257 individuals were examined. Of which 206 of them were acute stroke patients (aaa mean age 58.6610.6 years) and 51 were healthy persons, comparable by age and gender. DNA was successfully extracted from peripheral leucocytes in 206 acute stroke patients (94 female and 112 male) and 51 healthy persons (35 female and 16 male).

The genotype frequencies and allele rates for the AT1 gene A1166C polymorphism in the control group, all patients group and the patient subgroups are given in Table 2. There was no statistically significant difference in genotype frequencies between the control group and the patient group (*P* > 0.05).

**Table 2:** Distribution of genotypes and alleles according to stage of controls, total patients and patient subgroups

	Genotypes			Alleles		
		
	AA, n (%)	AC, n (%)		A, n (%)	C, n (%)	
Control	43 (84.3)	8 (15.7)	***P*** **<** **0.001**†**b**	94 (92.15)	8 (7.85)	***P*** **<** **0.001**†**d**
Patients	194(94.2)	12 (5.8)	***P*** **<** **0.001**†**b**	400 (97)	12 (3)	***P*** **<** **0.001**†**d**
	*P* > 0.05*a	*P* > 0.05*a		*P* > 0.05*c	*P* > 0.05*c	

	Genotypes		Alleles	
		
Stroke subtypes	AA, n (%)	AC, n (%)	A, n (%)	C, n (%)
Large vessel disease	46 (96)	2 (4)	94 (98)	2 (2)
Small vessel disease (lacunae)	56 (93)	4 (7)	116 (97)	4 (3)
Cardioembolism	29 (94)	2 (6)	60 (97)	2 (3)
Transient ischaemic attacks (TIA)	7 (78)	2 (22)	16 (89)	2 (11)
Other ischaemic strokes	10 (100)	0 (0)	20 (100)	0 (0)
Haemorrhage	46 (96)	2 (4)	94 (98)	2 (2)
	*P* > 0.05‡		*P* > 0.05‡	

* Two Proportions *z* test.

† One Proportion exact test, a: Control *versus* Patient in each Genotypes, b: AA *versus* AC in each groups, c: Control *versus* Patient in each Alleles, d: A *versus* C in each groups.

‡ Pearson Exact Chi-Square.

There was no statistically significant difference between the stroke subtypes (*P* > 0.05) in genotype and allele frequencies. Genotype and allele frequencies, however, showed a significant difference (*P* < 0.001) in the control and the patient groups.

Individual features of patients and controls are shown in Table 3 according to the AT1 gene A1166C polymorphism genotypes. There was no association between A1166C genotypes and individual features. However, in the heart disease control group, there was no association between the A1166C genotype of the AT1 gene and individual features (*P* = 0.09).

**Table 3:** Distribution of AT1 gene A1166C polymorphism genotypes according to individual features of controls and patients

Individual features	Groups	AA (%)	AC (%)	Statistic
Hypertension	Controls	92.3	7.7	*P = 0.003
	Patients	92	8	*P < 0.001
Smoking	Controls	87.5	12.5	*P = 0.035
	Patients	96	4	*P < 0.001
Alcohol use	Controls	0	0	†
	Patients	92.9	7.1	*P = 0.001
History of stroke	Controls	0	0	†
	Patients	85.7	14.3	*P < 0.001
Trauma	Controls	100	0	†
	Patients	95	5	*P < 0.001
Heart disease	Controls	77.8	22.2	*P = 0.09
	Patients	92.1	7.9	*P < 0.001
Vessel disease	Controls	0	0	†
	Patients	95.1	4.9	*P < 0.001
Diabetes	Controls	81.8	18.2	*P = 0.033
	Patients	93.9	6.1	*P < 0.001
Renal disease	Controls	0	0	†
	Patients	91.7	8.3	*P = 0.003

* One Proportion exact test.

† Could not be statistically analysed because of genotype quantity.

Some physiological and clinical parameters of patient and controls are presented in Table 4 according to genotype. There was no association between A1166C genotypes of the AT1 gene and these parameters.

**Table 4:** Distribution of AT1 gene A1166C polymorphism genotypes according to some physiological and clinical parameters of controls and patients

Parameters		AA *n* mean ± SD median (25th–75th) pctl	AC *n* mean ± SD median (25th–75th) pctl	Statistics
				AA-AC
Age (year)	Controls	*n* = 43 57.79 ± 8.29 59 (52–63)	*n* = 8 59.00 ± 11.14 59.5 (51.25–69.5)	*P = 0.584
	Patients	*n* = 194 62.78 ± 12.73 63.5(54–73)	*n* = 12 66.16 ± 11.92 70 (59–75)	*P = 0.274
Weight (kg)	Controls	*n* = 43 69.81 ± 11.47 69 (60–75)	*n* = 8 69.12 ± 7.29 71 (63.5–74.75)	*P = 0.732
	Patients	*n* = 194 72.04 ± 12.87 73 (65–80)	*n* = 12 72.00 ± 14.23 71 (65–84.75)	*P = 0.946
Systolic blood pressure (mmHg)	Controls	*n* = 43 124.37 ± 8.54 122 (120–130)	*n* = 8 118.75 ± 5.17 120 (115–123.75)	*P = 0.052
	Patients	*n* = 194 152.43 ± 33.27 141.5(130–171.5)	*n* = 12 151.66 ± 26.91 145 (125–180)	*P = 0.932
Diastolic blood pressure (mmHg)	Controls	*n* = 43 81.44 ± 7.48 80 (78–90)	*n* = 8 78.50 ± 4.20 80 (75.75–81.50)	*P = 0.277
	Patients	*n* = 194 88.89 ± 17.02 90 (80–100)	*n* = 12 87.50 ± 12.88 80 (80–97.5)	*P = 0.797
Creatinine (mg/dl)	Controls	*n* = 43 0.80 ± 0.21 0.77 (0.68–0.87)	*n* = 8 0.72 ± 0.09 0.75 (0.65–0.80)	*P = 0.288
	Patients	*n* = 194 0.99 ± 0.60 0.88(0.72–1.10)	*n* = 12 1.30 ± 0.76 1.02 (0.87–1.39)	*P = 0.056
High Density Lipoprotein (HDL-C) (mg/dl)	Controls	*n* = 43 50.18 ± 10.59 48 (41–56)	*n* = 8 48.25 ± 6.51 46 (42.5–55.25)	*P = 0.620
	Patients	*n* = 194 47.84 ± 15.29 48.5 (38–57)	*n* = 12 39.33 ± 13.69 41 (25.75–50.25)	*P = 0.070
Total cholesterol (mg/dl)	Controls	*n* = 43 200.37 ± 4.06 196 (164–228)	*n* = 8 183.87 ± 3.60 197.5 (159.5–211.25)	*P = 0.437
	Patients	*n* = 194 184.54 ± 5.13 180 (151.75–216.25)	*n* = 12 169.91 ± 6.30 153 (126.5–232.5)	*P = 0.164
Triglyceride (mg/dl)	Controls	*n* = 43 138.95 ± 61.12 129 (90–169)	*n* = 8 113.0 ± 46.78 110.5 (70.25–143.25)	*P = 0.254
	Patients	*n* = 194 100.13 ± 62.40 88.5 (57.75–133.25)	*n* = 12 84.50 ± 69.22 71 (37.5–102.75)	*P = 0.238

* Mann–Whitney U test, pctl: percentile.

The distribution of the A1166C polymorphism in the AT1 gene in patients and controls are given in Table 5 according to gender. There was no significant difference between the females and males of the control group.

**Table 5:** AT1 gene A1166C polymorphism genotypes according to gender in controls and patients

*n*		AA (*n* %)	AC (*n* %)	Statistics
Controls	Women 35	29 (82.9)	6 (17.1)	*P = 0.999
	Men 16	14 (87.5)	2 (12.5)	
Patients	Women 94	88 (93.6)	6 (6.4)	†*P* = 0.988
	Men 112	106 (94.6)	6 (5.4)	

* Fisher's Exact Test.

† Yates' Chi-Square.

## Discussion

Some recent studies have presented that there is the genetic predisposition to the development of stroke. However, some studies provide contradictory evidence, either in favour or not of a correlation between the A1166C polymorphism and stroke.

In this study, we analysed the distribution of AT1 gene A1166C polymorphism genotypes in acute stroke patients in Turkey to assess its possible role in the pathogenesis of acute stroke.

When we examined every patient and control in our study for their genotype of the AT1 gene at the A1166C position, we found the following: 84% AA and 16% AC for controls and 94% AA and 6% AC for patients. There was no statistically significant difference between the control group and the patient group in terms of genotype and allele frequency.

In two studies of 308 ischaemic stroke patients, Szolnoki *et al*. reported that the angiotensin II type 1 receptor A1166C polymorphism is associated with the development of small vessel ischaemic stroke 13 and increased risk of ischaemic stroke in hypertensive smokers 14. In a study of 215 ischaemic stroke patients, aaaRubattu *et al*. reported a predisposing role of an AT1 gene variant in the development of ischaemic stroke. In particular, the AT1 gene variant exerted a major impact on ischaemic stroke occurrence in the presence of hypertension 16. In a study of 152 type 2 diabetic patients with strokes, Zhang *et al*. reported a synergistic effect of the angiotensin II type 1 receptor A1166C and ACE I/D gene polymorphisms on the occurrence and development of stroke in type 2 diabetic patients in China 20.

Henskens *et al*. reported that AT1R A1166C polymorphism is significantly associated with subcortical white matter lesions (WMLs). In addition, they have reported that when using the AA genotype as the reference category, the CC genotype of the AT1R A1166C polymorphism is inversely associated with the volume of subcortical WMLs 19.

Mollsten *et al*. have determined that individuals with the AA genotype of the AT1R gene are at increased risk of ischaemic stroke compared with those with the AC and CC genotypes. They have reported that A1166C polymorphism in the angiotensin II receptor gene is associated with ischaemic stroke 21.

Takami *et al*. reported that AT1R polymorphism was significantly associated with the number of lacunae in the basal ganglia and whole brain regions and with periventricular hyperintensity grade in the younger population. They have reported that AT1 polymorphisms are independent genetic risk factors for lacunar infarction 22.

Brenner *et al* have determined 1166 AA genotype frequency as 49.2%, AC genotype frequency as 41.6%, CC genotype frequency as 9.2% for patients; and AA genotype frequency as 56.0%, AC genotype frequency as 34.4% and CC genotype frequency as 9.6% for control group. They have reported that 1166C allele is a weak association of brain infarction 3.

Sipahi *et al*. have determined 1166 AA genotype frequency as 58%, AC genotype frequency as 34.6%, CC genotype frequency as 7.4% for patients; and AA genotype frequency as 60.1%, AC genotype frequency as 35.7% and CC genotype frequency as 4.2% for control group. Similar to our findings, they have also reported no difference among the stroke subgroups regarding the distribution of AT1R (A1166C) polymorphisms 15.

In a study of 129 ischaemic stroke patients, aaaMizuno *et al*. reported that the angiotensin II type 1 receptor A1166C polymorphism showed no significant effect on ischaemic stroke, similar to our findings in this study. However, the ACE polymorphism has a role in lacunar infarction that is independent from hypertension 2.

Wiklund *et al*. have determined 1166 AA genotype frequency as 59%, AC genotype frequency as 34%, CC genotype frequency as 17%, for ischaemic stroke patients; 1166 AA genotype frequency as 50%, AC genotype frequency as 39%, CC genotype frequency as 12%, for haemorrhagic stroke patients and AA genotype frequency as 50%, AC genotype frequency as 42% and TT genotype frequency as 7% for control group. They have reported that individuals carrying the AA genotype of the angiotensin II type 1 receptor A1166C polymorphism to be at somewhat higher risk of future stroke as compared with the AC and CC genotypes 1.

In conclusion, although there are various population studies showing significant association between AT1 gene A1166C polymorphism and susceptibility to stroke, our findings suggest that the AT1 gene A1166C polymorphism is not associated with susceptibility to acute stroke in Turkish population that might be due to particular genetic structure of Turkish population. It also clarifies a causality role to polymorphisms for the multifactorial nature of stroke pathogenesis, in which many genetic variants could contribute, together with environmental and the interactions of other genes. As a result, further studies in larger populations are required to clarify the interactions of the A1166C polymorphism with stroke due to the different findings from various populations.
